# Application and characterization of crude fungal lipases used to degrade fat and oil wastes

**DOI:** 10.1038/s41598-021-98927-4

**Published:** 2021-10-04

**Authors:** Amira Hassan Alabdalall, Norah A. Al-Anazi, Lena A. Aldakheel, Fatma H. I. Amer, Fatimah A. Aldakheel, Ibtisam M. Ababutain, Azzah I. Alghamdi, Eida M. Al-Khaldi

**Affiliations:** 1 Department of Biology, College of Science, Imam Abdulrahman Bin Faisal University, P.O.Box 1982, Dammam, Saudi Arabia; 2 Family and Community Medicine Department, College of Medicine, Imam Abdulrahman Bin Faisal University, Dammam, Saudi Arabia; 3 Basic & Applied Scientific Research Center (BASRC), Imam Abdulrahman Bin Faisal University, P.O. Box 1982, Dammam, 31441 Saudi Arabia

**Keywords:** Biochemistry, Biological techniques, Biotechnology, Chemical biology, Microbiology

## Abstract

*Aspergillus niger* MH078571.1 and *A. niger* MH079049.1 were identified previously as the two highest *Aspergillus niger* strains producing lipase. Biochemical characterizations of lipase activity and stability for these two strains were examined and revealed that the optimal temperature is 45 °C at pH 8for *A. niger* MH078571.1 and 55 °C for MH079049.1. The lipase production of both strains was studied on medium contains waste oil, as a cheap source to reduce the industrial cost, showed that the optimal incubation period for the enzyme production is 3 days. Moreover, an experiment on lipase activates in organic solvents demonstrated that 50% of acetone is the best solvent for the two strains. In the presence of surfactants, 0.1% of tween 80 surfactant showed the best lipase activities. Furthermore, Mg^2+^ and Zn^2+^ ions enhanced the lipase activity of *A. niger* MH078571.1, while Na^2+^ and Cu^2+^ enhanced the enzyme activity of *A. niger* MH079049.1. Lipase activity was also tested for industrial applications such as integrating it with different detergents. Maximum lipase activity was obtained with 1% of Omo as a powder detergent for both strains. In liquid detergent, 0.1% of Fairy showed maximum lipase activity in *A. niger* MH078571.1, while the lipase in *A. niger* MH079049.1 was more effective in 1% of Lux. Moreover, the degradation of natural animal fat with crude enzyme was tested using chicken and sheep fats. The results showed that more than 90% of fats degraded after 5 days of the incubation period.

## Introduction

The reversible catalyzation of triglycerides into fatty acid is done by the lipase enzyme.

Some lipases are acting on both the transesterification and enantioselective hydrolysis reactions^1^. The interest in studying lipase production has increased over the past several years due to their excellent catalytic properties^2^, and diverse industrial applications, including detergents, esterification, pharmaceuticals, and production of biodiesel^3^. Lipase enzyme presents widely in bacteria, yeasts, and fungi^4^.

The demand for microbial lipase production increases globally based on the type of source. It generates significant revenues as it is expected to exceed USD 797.7 million by 2025^5^.

There is a fast-growing in the lipase market as it is likely that the liquid segment will represent the highest growth rate in terms of revenue in the lipase market. Moreover, the powder segment is expected to record a Compound annual growth rate (CAGR) of 5.4% until 2026^6^.

It is proposed that by 2024, detergent applications may observe significant gains and surpass USD 2 billion in value^7^. In 2017, the largest market for industrial enzymes was the food and beverages with an estimated value of USD 1.4 billion in 2017; this was followed by biofuels and detergents with an estimated value of USD 969.3 million and USD 754.4 million, respectively. Industrial enzymes in biofuels are expected the fastest-growing segment with a CAGR of 7.3% until 2024 à Dublin^8^.

The importance of enzymes is ever-rising, particularly microbial lipases holding great industrial worth owing to their potential to catalyze a diverse array of chemical reactions in aqueous and nonaqueous settings. The international lipase market is anticipated to cross USD 797.7 million till 2025, rising at a 6.2% compound annual growth rate from 2017 to 2025.

Enzymes from animals, plants, and microorganisms are commercially available, with the microbial origin being the main source presenting over 50% of the industrial enzymes^9^. One-third of the total worldwide industrial enzyme incorporated into detergent^10^. With thermal stability being an essential requirement for commercial enzymes^11^, various temperatures should not affect the lipase enzymatic reaction. While the lipase degrades fat at low temperatures, higher temperatures increase fat degradation rates in the present of lipase.

The major industrial applications of lipase enzyme were in the manufacturing of detergents due to its remarkable ability in removing oil stains from fabrics. However, in order to use lipase, it has to be thermally stable, alkaline-tolerant, and could degrade various lipid structures. Using enzymes instead of the traditional chemical detergents is more effective; because it saves energy by acting at lower temperatures, is environment friendly, has no threat to aquatic life, and has no adverse effect on wastewater treatment processes^12^.

Saisubramanian et al.^13^ reported that the application of *A. niger*'s lipase as an additive in laundry detergents showed an increased instability in the presence of SDS, Tween 80 and all commercial detergents. The optimum conditions in Saisubramanian study were 1% for commercial detergents, 75 units of lipase, a pH 9.5, and a washing temperature at 25 °C. Under these conditions, 33% of the olive oil stains in the cotton fabric were removed.

*Aspergillus* genus was used before in the detergent industry, where a purified lipase from *A. carneus* showed a promising finding. The *A. carneus* lipase has an optimum temperature of 37 °C and pH 9. The study suggested that the presence of many detergents stimulated its activity^14^. Thermal and alkaline lipase produced by *Talaromyces thermophilus* showed significant resistance to alkaline pH, intermittency, and high tolerability with many surfactants, oxidation, and commercial washing materials. Thus, this enzyme can be considered a promising and satisfying candidate for the industrial application of the cleaning process^15^. Washing is generally performed in alkaline media, and lipase favors this condition, such as the lipase derived from *A. oryzae*^16^.

Other applications of detergents are in dishwashing, in bleaching formulation^17^, lipolysis of lipids in dry cleaning solvents^18^, liquid skin cleaner^19^, and contact lens cleaning^20^. Using lipase along with oxidoreductases in washing, degreasing, and water regeneration allows smaller amounts of surfactants and operate at low temperatures^21^. When lipase has been integrated into detergents, it promotes better cleaning and prevents scaling. More recently, lipase extracted from *Rhizopus nigricans* has shown excess lipid analytical activity and natural emulsification activity, indicating the highest rate of production of surfactants^22^.

The objective of this study is (A) to determine the biochemical characterizations of lipase activity and stability for *A. niger* MH078571.1 and *A. niger* MH079049.1; (B) to test the lipase activity for industrial applications such as (i) integrating the enzyme with powder and liquid detergents (ii) evaluating its ability to remove fats and (ii) eliminating different oil stains from fabric.

## Materials and methods

### The source of the fungal strains isolates

The two highest lipase-producer strains identified by partial 18S rDNA sequencing as *Aspergillus niger* (GenBank Accession No*.* MH078571.1 and MH079049.1) were obtained from a previous study^23^ at Institute for Research and Medical Consultations (IRMC) in Imam Abdulrahman Bin Faisal University, Dammam, Saudi Arabia. Pure cultures of these two fungi were maintained on Potato Dextrose agar slants and stored at 4 °C.

### Lipase production by submerged fermentation

The lipase produced by both strains (*A. niger* MH078571.1 and *A. niger* MH079049.1) were isolated using Tween 80 broth, following the protocol in Ayinla et al.^24^. In brief, a fungal conidial (3 × 10^7^ spores/ml) suspended in a mineral salt solution were used to inoculate the two fungal strains separately. An aliquot (2 ml) of each of these suspensions were inoculated into sterile mineral salt solution media. Then, both sterile media were incubated for 5 days at 25 ± 2 °C on a rotary shaker at 200 rpm. After the 5 days incubation, Whatman’s filter paper no.1 was used to filter the fungal growth of each strain. The Whatman’s filter papers containing the filtered fungal cultures were centrifuged at 1200 rpm for 30 min at 4 °C. After centrifugation, two clear supernatants were obtained containing the crude lipase enzyme of each strain.

### Lipase production assay using *p*-Nitrophenyl Palmitate (pNPP) as a substrate

Since lipase claves the *p*-Nitrophenyl Palmitate (pNPP) substrate, the common pNPP hydrolysis method was used to assess the lipase production. Following Oliveira et al.^25^ procedure, for each strain, an aliquot (100 µl) of each clear supernatant was added to 900 µl of a reaction mixture with the following composition: 800 µl of 0.25% polyvinyl alcohol solution at pH 6.5 and 100 µl of pNPP solution (3 mM) in isopropanol. The reaction mixtures were incubated for 15 min at 30 °C. After incubation, the reaction was terminated by adding 500 µl of HCl (3 mM) into the mixtures (1:1 v/v). An aliquot (500 µl) of the final two mixtures were added into a 1 ml of NaOH (2 mM). Spectrophotometer (Spectro UV–Vis Double) was used to measure the lipase production at 410 nm. To measure the enzyme activity, a standard curve was used as described previously by Oliveira et al.^25^. One unit of lipase activity was defined as the amount of lipase required to release 1 µM of pNPP in one minute, under the specified conditions.

### Characteristic for the lipase

In pH 8, the optimum temperature according to our previoud study^23^ was determined as 45 and 55 for *A. niger* MH078571.1 and *A. niger* MH079049.1, respectively. Sethi et al.^26^ procedure was followed to determine the effective temperature for the lipase enzyme activity and stability.

The clear supernatants were used with these factors to identify the optimum conditions for the lipase activity and its stability to be used in various industrial and commercial applications: (A) various temperatures ranging from 25 to 70 °C; (B) different pH ranging from 1 to 10 for 24 h^27^; (C) various concentrations of enzyme with organic solvents (methanol, ethanol, ispropanol, butanol, acetone)^28^; (D) various concentrations of surfactant solutions [tween 80, tween 20, sodium dodecyl sulfate (SDS)]^29^; and (E) various metal ions (CaCl_2_, NaCl, KCl, K_4_, NH_4_Cl, MgSo_4_, CuSo_4_, ZnSo_4_, EDTA)^27^.

### Determination of lipase efficiency

After determining the enzyme optimum condition, the clear supernatants, which contain the crude fungal lipase, were tested for its efficiency in (A) detergent; (B) removing oil stains from fabrics; (C) degrading natural animal fat:

#### Detergent

The clear supernatants were tested for its efficiency with both liquid (Fairy, pandah, dac, lux, perial) and powder (Tide, Arial, pandah, Persil, Omo) detergents following Bacha et al.^30^ protocol. In brief, the clear supernatants were mixed with liquid and/or powder detergents at 0.1% and 1% concentrations (v:v or w/v) for 30 min at 35 °C. The enzyme activity was then assessed through the Spectrophotometer at 410 nm absorbance.

#### Removing oil stains

The clear supernatants were also tested for their efficiency in removing oil stains from fabrics following Das et al.^31^ protocol. In brief, a polycotton fabric was cut into pieces (3 cm × 2 cm) and each piece was stained with two drops of either a car, frying fish, or chocolate oil. The fabric pieces then were allowed to dry and placed on 100 ml flasks with four different treatments.

The four treatments were: (a) water (100 ml), (b) water with 1% (w/v) detergent (99 ml + 1 ml), (c) water with lipase (99 ml + 1 ml), (d) water with lipase and detergent (98 ml + 1 ml + 1 ml). Each treatment was done in duplicate, once with cold water (25 ± 2 °C) and once with hot water (65 ± 2 °C). All treatments were incubated and gently agitated for 30 min. Fabric pieces were then removed, dried, and examined for the present of oil residual.

To estimate the amount of free fatty acids released during each treatment, titrimetric assay was used following Dayanandan et al.^32^ procedure.

#### Degradation of natural animal fat

The clear supernatants were tested for its efficiency in degrading natural animal fat following Uppada et al.^33^ protocol. In brief, pieces of chicken and sheep fats (2.5 g) were autoclaved and placed in tubes before adding the clear supernatants. The tubes then were incubated at their optimum temperature (45 °C and 55 °C respectively for *A. niger* MH078571.1 *and A. niger* MH079049.1) for 24, 48, 72, 96, 120, and 144 h. After incubation, fat pieces were weighted, and the enzyme activity was measured following the protocol in Oliveira et al.^25^.

### Statistical analysis

All experiments were carried out in triplicates (n = 3) and the average values were used. The ± sign and the error bars represent the standard deviation of the mean. For each individual experiment, one-way ANOVA was calculated using the SPSS 16.0 software. The least significant differences (LSD) were examined by analyzing variance (ANOVA) using SPSS software version 23^34^.

### Ethical approval

This article does not contain any studies with human participants or animals performed by any of the authors.

## Results

### Characteristic for the lipase

The optimum conditions of enzyme activity and stability (temperature, pH, organic solvents, surfactants, and mineral ions) were studied to determine the possibility of enzyme applications. Therefore, both isolates *A. niger* MH078571.1 and *A. niger* MH079049.1 were cultured under the following optimal conditions to obtain the highest activity of the crude fungal lipase:

#### Effect of temperature

Generally, the high temperature leads to the acceleration of chemical reactions. However, it must be considered that enzymes are proteins and can be damaged by very high temperature or when exposed to heat for long time. Each enzyme has an ideal temperature range where it performs the best; and an increase or decrease in this ideal temperature range would affect that enzyme performance negatively.

The highest activity of lipase production by *A. niger* MH078571.1 was at 45 °C, where the highest lipase performance obtained after an hour of incubation (794.23 U/ml) (Supplementary Table S1). After 24 h of incubation, the effect of lipase activity at 45 °C lost only 16% (Supplementary Figure S1). For *A. niger* MH079049.1 however, the highest activity of lipase production was at 55 °C, where the highest lipase performance obtained after an hour of incubation (796.92 U/ml) (Supplementary Table S1). After 24 h of incubation, the efficacy of lipase activity at 55 °C lost only 14% (Supplementary Figure S1).

#### Effect of pH

Each enzyme has an ideal pH that it become the most effective at; and decreases or increases in that pH would negatively affect the enzyme performance. Lipase was more effective and stable at an alkaline pH of 8 for both isolates. After an hour of incubation, the lipase activity of *A. niger* MH078571.1 and *A. niger* MH079049.1 were 795.39 and 795.77 U/ml, respectively. (Supplementary Table S2). After 24 h of incubation, *A. niger* MH078571.1 strain lost 14% of its lipase activity at pH 8 while *A. niger* MH079049.1 strain lost 20% of its lipase activity at the same pH (Supplementary Figure S2).

#### Effect of organic solvent

Organic solvents have a significant role in influencing the activity of enzymes and their effectiveness. The effectiveness of an enzyme in the presence of these solvents is essential and necessary in industrial processes. It has been noticed that the best solvent for lipase was acetone, followed by methanol and butanol while isopropanol and ethanol were the least energizing of the enzyme.

The organic solvents were used at two different concentrations (50% and 100%) to test the optimal activity of lipase for both isolates. The 50% organic solvents concentration showed higher enzyme activity. Of the organic solvents, the effectiveness of lipase activity reached 96.5% for *A. niger* MH078571.1 and 93% for *A. niger* MH079049.1 in the presence of acetone at 50% concentration. However, when isopropanol solvent was used, the two strains’ enzyme activity was the least effective (Supplementary Table S3; Supplementary Figure S3).

#### Effect of surfactants

The surfactants were used at two different concentrations of 0.1% and 1% to test the optimal lipase activity in both isolates. The lipase activity for both isolates was best at 0.1% concentration*.* Of the surfactants, the effectiveness of lipase activity reached 103.42% for *A. niger* MH078571.1 and 112.4% for *A. niger* MH079049.1 in the presence of tween 80 at 0.1% concentration. However, the lipase activity in the presence of SDS was the least effective at 0.1% concentration (Supplementary Table S4; Supplementary Figure S4).

#### Effect of ions

Ions bind to enzymes and activate them. Ions binding to enzyme could improve or inhibit the enzyme activity. For this reason, eight ions were used at two different concentrations (0.1% and 1%) to test the optimal activity of lipase for both isolates. The 0.1% ions concentration showed higher enzyme activity. Of the eight ions, the effectiveness of lipase activity reached 102% for *A. niger* MH078571.1 isolate in the present of both manganese sulfate and zinc sulfate. For *A. niger* MH079049.1 isolate however. the sodium chloride at 0.1% concentration showed the highest lipase activity of 105% (Supplementary Table S5; Supplementary Figure S5).

### Determination of the optimal storage temperature for lipase

The relation between lipase activity and storage temperature was depicted in Supplementary Table S6 and Supplementary Figures S6. The best storage temperature for *A. niger* MH078571.1 was at – 80 °C, where the enzyme maintained more than 75.31% of its activity after four weeks, while 25 °C is the least efficient as the enzyme lost more than 95% of its activity after four weeks. In the case of *A. niger* MH.079049.1, the enzyme has maintained more than 73.04% of its activity at – 80 °C after 4 weeks. This enzyme has lost about 98% of its effectiveness in the same storage conditions at 25 °C after four weeks (Supplementary Table S6).

### Determine the efficiency of lipase activity in natural oil waste

Oil is one of the primary and most important factors in stimulating enzyme production. Therefore, different types of oil residues present in nature were used for each of the two isolates *A. niger* MH078571.1 and *A. niger* MH.079049.1 (Supplementary Table S7). Although the potato frying oil had the highest lipase activity in the first 3 days of the experiment, the results showed that vehicle oil maintained its high activity through the 15 days. The enzymatic unit in the presence of fish oil has the lowest activity among the others.

The best time for enzyme production was also tested, and 3 days were the highest enzyme production in all types of oils. The enzyme productivity was decreased with an increase in the number of days.

### Determination of lipase efficiency

#### Detergent

Several powder and liquid detergents were used at two different concentrations of 0.1% and 1% to test the optimal activity of lipase in *A. niger MH078571.1 and A. niger* MH.079049.1.

The lipase activity for both isolates was best at 1% concentration with powder detergents*.* Of the powder detergents, the effectiveness of lipase activity reached 91.07% for *A. niger* MH078571.1 with 1% Omo and 95.08% for *A. niger* MH.079049.1 with 1% Persial (Supplementary Table S8).

In the presence of liquid detergents, however, the effectiveness of lipase activity reached 92.89% for *A. niger* MH078571.1 with 0.1% Fairy and 97.54% for *A. niger* MH.079049.1 with 1% Lux (Supplementary Table S9; Supplementary Figures S7–S8).

#### Removing oil stains

To determine the efficacy of lipase in removing oil stains from cotton fabrics, four treatments were compared (Table 1 and Supplementary Figure S9). It has been observed that the enzyme activity for both isolates was better at high temperatures (65 °C) and lower at low temperatures (25 °C).

**Table 1:** The determination of lipase efficiency and activity of both *A. niger* isolates in removing oil stain from polycotton fabric.

Isolates	*A. niger* MH078571		*A. niger* MH079049	
Treatment	Cold activity (U/ml) 25 °C*	Hot activity (U/ml) 65 °C*	Cold activity (U/ml) 25 °C*	Hot activity (U/ml) 65 °C*
**Vehicles oil**				
Water	9.528	19.789	9.528	19.789
Water + detergent	132.67	132.67	132.67	132.67
Water + lipase	240.41	322.50	245.54	317.37
Water + lipase + detergent	327.63	378.94	337.89	394.33
**Fish oil**				
Water	19.79	40.312	19.789	40.312
Water + detergent	122.40	142.93	122.4	142.93
Water + lipase	245.54	353.29	245.54	301.98
Water + lipase + detergent	358.42	430.25	348.16	425.12
**Chocolate**				
Water	14.659	35.181	14.66	35.181
Water + detergent	76.227	91.619	76.227	91.619
Water + lipase	199.36	230.15	204.5	240.41
Water + lipase + detergent	230.15	266.06	225.02	301.98

*Averages of three replicates.

**Figure 1:**
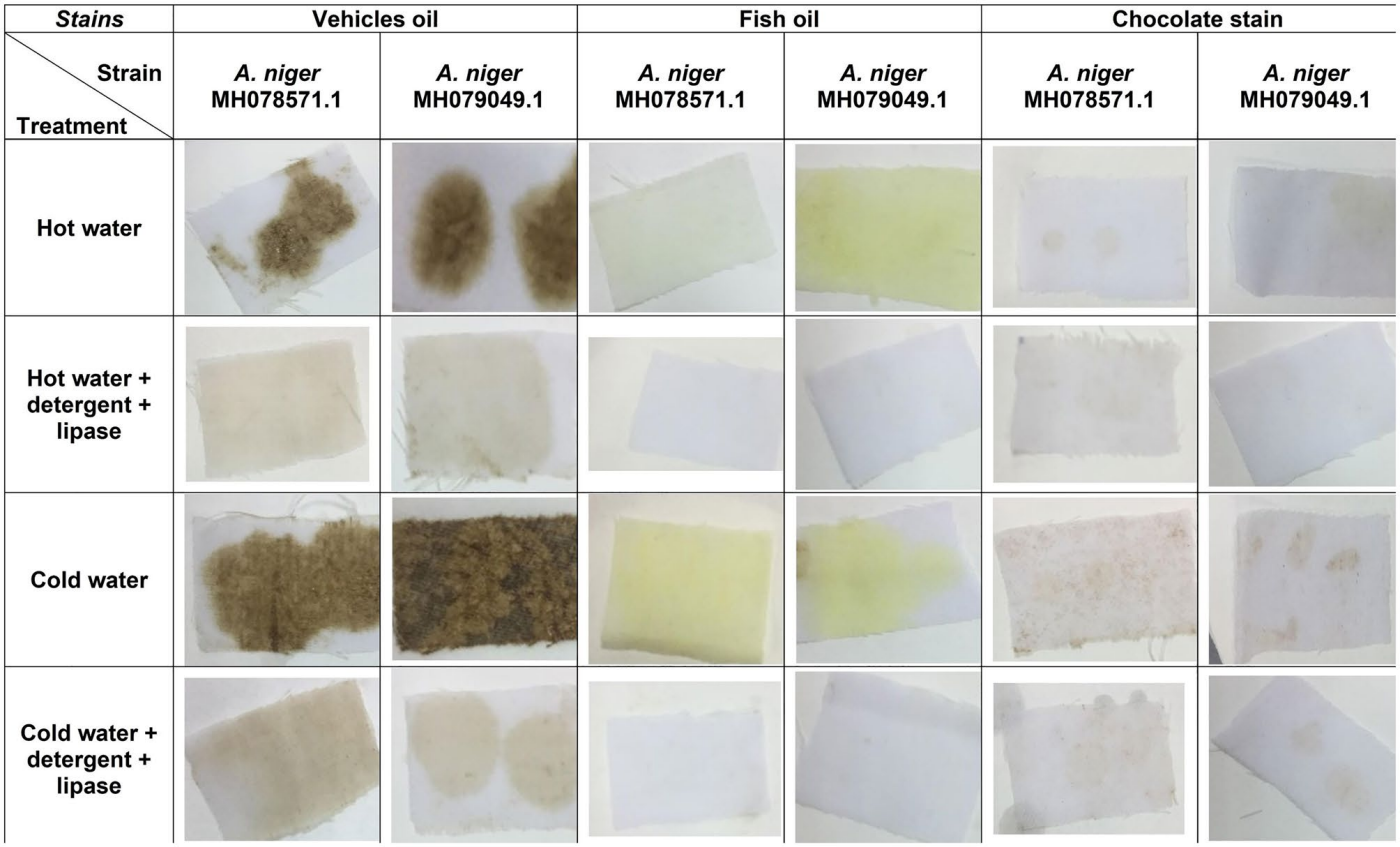
The effect lipase enzyme from both *A. niger* MH078571.1 and *A. niger* MH079049.1 on removing vehicles oil stain, fish oil stain, and chocolate stain.

The enzyme's effectiveness in removing stains at 65 °C becomes more evident when noticing the figures, as more than 80% of the stain was eliminated when treated at a high temperature using the enzyme and the detergent together (Fig. 1, Supplementary Fig. S10). In contrast, at 65 °C when the cotton fabric treated with detergent alone, only about 45% of the oil stains were removed; and when treated with the enzyme alone, approximately 50% of the oil stains were removed.

From the three oil types (vehicle, fish, and chocolate oil), the fish oil had the highest enzyme activity for both isolates in the presence of detergent and enzyme together at 65 °C.

#### Degradation of natural animal fat

The lipase enzyme can hydrolyze triglycerides into fatty acids and glycerol. This enzyme ability was adopted to degrade animal fats (chicken and sheep). Lipase enzyme from two isolates were able to degrade more than 90% of the chicken fat and more than 85% of sheep fat (Tables 2, 3, Supplementary Figures S11, S12). The lipase activity was high in the first 3 days, where more than half of the degradation process took place.

**Table 2:** The determination of lipase efficiency and activity of both *A. niger* isolates in degrading chicken fat.

Isolates	*A. niger MH078571*			*A. niger MH079049*		
	Weight*	Absorbance*	Lipase activity (U/ml)*	Weight*	Absorbance*	Lipase activity (U/ml)*
Control	2.5	1.22	773.85	2.5	1.26	796.92
1st day	1.2	1.1	681.54	0.964	1.147	717.69
2nd day	0.635	1.064	653.85	0.749	1.023	622.31
3rd day	0.47	0.894	523.08	0.526	0.847	486.92
4th day	0.366	0.764	423.08	0.489	0.565	270.00
5th day	0.28	0.43	166.15	0.33	0.417	156.15
6th day	0.242	0.22	4.62	0.289	0.219	3.85

*Averages of three replicates.

**Table 3:** The determination of lipase efficiency and activity of both *A. niger* isolates in degrading sheep fat.

Isolates	*A. niger* MH078571			*A. niger* MH079049		
	Weight*	Absorbance*	Lipase activity (U/ml)*	Weight*	Absorbance*	Lipase activity (U/ml)*
Control	2.5	1.22	773.85	2.5	1.25	796.92
1st day	2.15	1.14	712.31	2.07	1.06	650.77
2nd day	0.915	1.025	623.85	1.188	0.942	560.00
3rd day	0.852	0.943	560.77	1.003	0.794	446.15
4th day	0.647	0.696	370.77	0.627	0.653	337.69
5th day	0.41	0.358	110.77	0.383	0.428	164.62
6th day	0.36	0.23	12.31	0.32	0.242	21.54

*Averages of three replicates.

## Discussion

Lipase is one of the most essential biocatalysts. It performs reactions in the aqueous and non-aqueous medium. It stimulates the aqueous degradation of triglycerides to glycerol and fatty acids. As a result of lipase’s chemical and mechanical properties, efforts were focused on it in the field of scientific and industrial research. Lipase enzymes are found in most organisms, such as animals, plants, yeasts, fungi, and bacteria. Microbial lipase enzymes have gained special industrial attention due to their ability to maintain their activity under extreme temperatures, pH and organic solvents, and chemical conditions. The fungal lipase emerges as a key enzymatic source due to its catalytic activity, low cost of production, and relative ease in genetic manipulation^35^.

**Figure 2:**
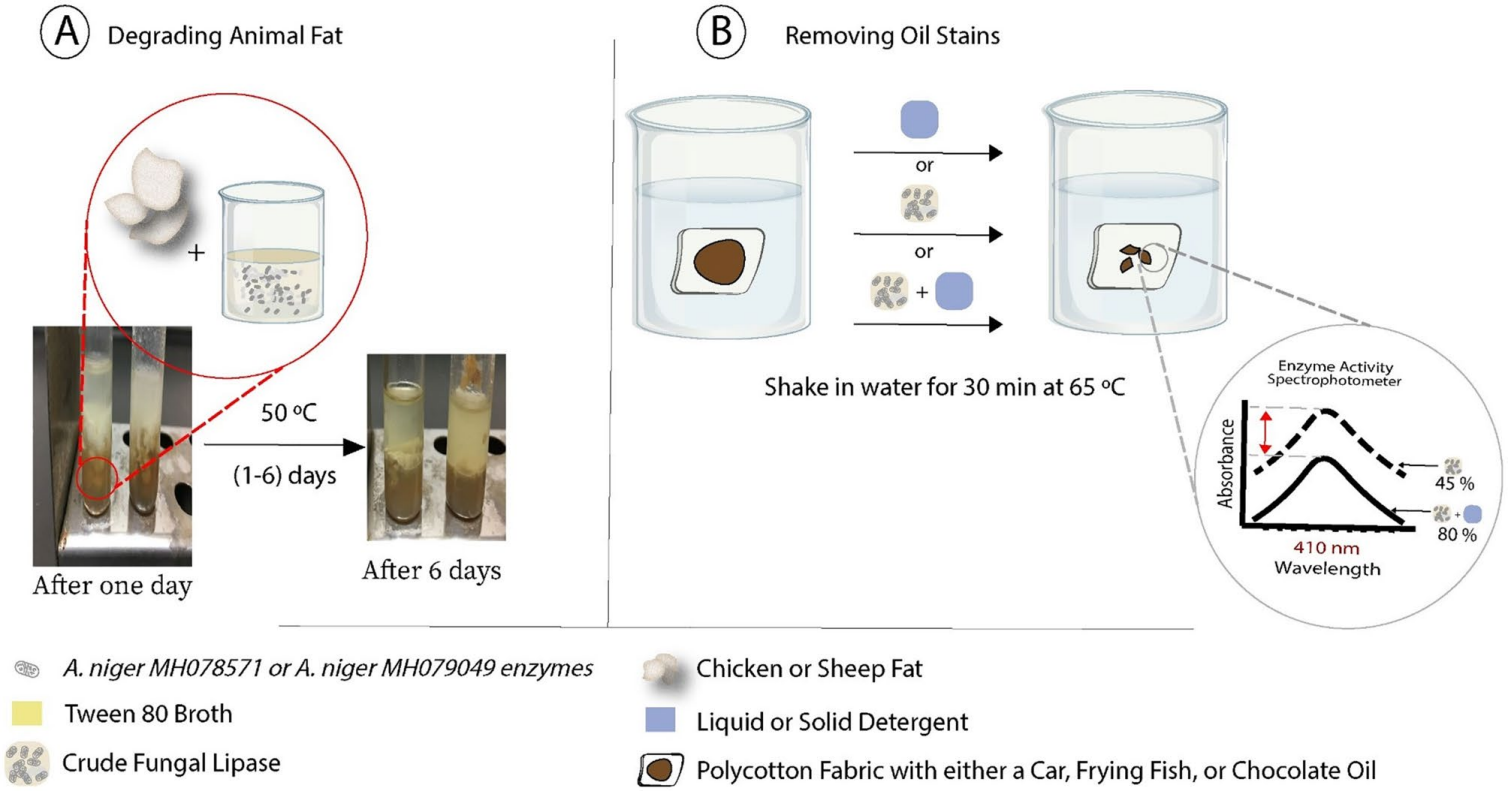
A summary of the main steps conducted to validate the industrial application of using lipase enzymes produced by *A. niger* MH078571.1, and *A. niger* MH079049.1 to (**A**) degrade animal fat and (**B**) remove oil wastes^36^.

The two most productive isolates for lipase in Aspergillus genus are *A. niger* MH078571.1, and *A. niger* MH079049.1. Both isolates were chosen to study their enzyme physiological and biochemical properties and their applicability in various industrial applications (Fig. 2).

The enzyme tolerance to high temperatures and the stability of its activity is clearly beneficial in industrial processes. It contributes to raising the reaction rate and process yield by increasing the solubility of the reaction materials and products. It also displaces the balance in endothermic reactions and reduce bacterial contamination^37^.

The results showed that the enzyme activity’s temperature is the highest and more stable after 24 h of incubation is 45 °C for the enzyme produced by *A. niger* MH078571.1 and at 55 °C for the enzyme produced by *A. niger* MH079049.1*.* This would allow the enzyme in various commercial industries to be carried out at high temperatures (< 70 °C), such as lipid analysis, esterification, and Biodiesel production. Whereas the two enzymes lost most of their activity at 70 °C due to the denaturation of enzymatic proteins at high temperatures^38^.

These results were consistent with Falony et al.^39^, where they stated that lipase produced by *A. niger* had the optimum activity at 55 °C. Namboodiri et al.^40^ reported that the maximum activity of the producing lipase by *Humicola lanuginose* was at a temperature of 45 °C. They also reported that the thermal stability of the lipase enzyme was found at the temperature is 50 °C^30^.

Whereas most lipase activity produced by Aspergilli are at around 40 °C, for example, *A. niger* NCIM1207 lost 52% of the initial activity after an hour incubation at 50 °C^41^. Sundar and Kumaresapillai^42^ reported that the maximum enzymatic activity of lipase produced by *A. niger* is 40 °C; as 50 °C caused the enzyme to be denatured. Rashma and Shanmugam^43^ stated that the optimum temperature was 27 °C for the enzyme produced by *A. Brasiliensis*.

The optimum pH of the lipase activity produced by both strains under pH 8 was considered, where the enzyme is more active and stable over 24 h, and where the alkali and endothermic high temperature is very attractive to produce biodiesel and biopolymers in addition to their potential use in the production of chemicals Agricultural, cosmetic, detergent, flavoring and pharmaceutical preparations, and thus the lipase enzyme extracted from the two strains could be *A. niger MH078571.1*, and *A. niger MH079049.1* the ideal candidate for various industrial and biotechnology applications^44^.

The results of the current study on enzyme activity at pH 8 were consistent with Malekabadia et al.^29^, while Cruege and Crueger^45^ stated that the optimum pH for a lipase enzyme was at 6. Falony et al.^39^ and Rashma and Shanmugam^43^ stated that the optimal activity of the enzyme is at pH 7, while^41^ reported that the activity of the enzyme lipase and its stability in the acidic environment at pH 3.

The lipase enzyme activity in various industrial processes depends significantly on the extent of the enzyme's tolerance, and its activity in the presence of different organic solvents. The activation of the lipase can be clarified by the interaction of these organic solvents with the amino acid residues present in the cap that protects the catalytic site in the enzyme protein. Thus, maintaining the lipase is in its apparent state retains its elasticity, which facilitates its transportation to the active site and the response of the active site movement to the treatment of the reaction^46^.

The results of the study confirmed the lipase enzyme ability, produced by the two strains *A. niger* MH078571.1 and *A. niger* MH079049.1, to withstand high concentrations (50%) of different organic solvents; whereby the enzyme maintained 96.5% and 93.1% of their activity in the presence of 50% of acetone. These results were consistent with the results of^14,30^ at the same focus, while^47,48^ that the lipase lost most of its activity when incubated with various organic solvents.

Organic solvents can strongly affect living cells' integrity and stability by binding to the cell membrane. Binding to cell membrane might disrupt its permeability leading to cellular metabolism damages, growth inhibition, and cell death^49^. In our experiment, we noticed that Lipase lost a large part (about 65%) of its efficacy in a 100% concentration of ethanol and acetone. This happens because the presence of organic solvents may cause drying by removing the water molecules from the enzyme circumference, which negatively affects its efficacy. Moreover, all organic solvents may cause denaturation of the amino acid residues present in an enzyme^31^. However, despite all these worse effects of organic solvents in living cells, there are organic solvent-tolerant bacteria capable of thriving in the presence of these toxic solvents^50^. The hydrolase-catalyzed perhydrolysis proceeds better in the present of organic solvent because of lower nucleophilic competition (H_2_O/H_2_O_2_) in these media.

Substances that affect surfactants are influences on enzyme activity in the industry, especially detergents. During washing, lipase enzymes must withstand useful cleaning materials in the presence of various surfactants along with their temperature and pH stability^51^.

In the presence of tween 80 at a concentration of 0.1%, it had a catalytic enzyme activity effect. The activity of the two enzymes increased by 103.4% and 112.4% for both enzyme strains *A. niger* MH078571.1 and *A. niger* MH079049.1*,* respectively. It is suggested that these abhorrent factors Water binds to the structure of the enzyme and changes occur in the formation of the enzyme, which increases the effective access to the substrate and increases the enzymatic activity gives promising advantages in the field of detergents^52^.

Bacha, et al.^30^, Das et al.^31^ and Malekabadia et al.^29^ all stated that tween 80 surfactant stimulated the enzymatic activity which is consistent with the obtained result. Zheng et al.^1^ and Sharma and Kanwar^49^ all supported the obtained results of SDS and tween 20 surfactants to have an inhibitory effect on enzyme activity. The inhibitory effect of these surfactants may be caused by disrupting the surfactants’ main structures, which corrupt enzyme activity^53^.

Dandavate et al.^54^ found that SDS and tween 20 both have a stimulating effect on enzyme activity, while the enzyme maintained 100% activity in the presence of surfactants, i.e. they did not affect the enzyme activity^55^.

Enzymes require metallic ions as common agents in various metabolic pathways. The results showed a slight increase on enzyme activity in the presence of both Zn^+^ and Mg^+^ at a concentration of 0.1% of 102% and in the presence of sodium 0.1% Na^+^ of 104.9% for the two enzymes produced by strains *A. niger* MH078571.1 and *A niger* MH079049.1, respectively Sahoo et al.^56^ also obtained the catalytic result of the enzyme in the presence of the same ions, while Yang et al.^57^ stated that magnesium has an inhibitory effect on enzymatic activity.

Concentration gave 1% an inhibitory effect of the enzyme in all the tested elements, while in concentration 0.1%, the effect of K^+^ and EDTA was the most inhibiting on the enzyme produced by *A. niger* MH078571.1, while Ca^+^ and EDTA was the most inhibiting of the enzyme produced by *A. niger* MH079049.1*.* The reason may be that these mineral ions bind to lipase in inactive sites instead of active sites, which reduces enzyme activity and efficacy^31^, while^1^ for EDTA has a catalytic role on the lipase enzyme.

After studying the physiological and biochemical properties of the enzyme and knowing the optimal conditions in which the enzyme is more active and stable, the optimum temperature was checked to maintain and stabilize the enzymatic activity as long as possible. For use in industrial applications later, the optimum degree for maintaining the raw enzyme was − 80%. The enzyme produced by the *A. niger* strain MH078571.1 sustained 75.31% of its activity, while the enzyme produced by *A. niger MH079049.1* retained 73.04% of its activity, and Souza et al.^28^ all obtained similar results, as the enzyme maintained 90% and 80% of its activity, respectively.

The possibility of producing lipase enzyme from the two strains from oily waste was studied for the oils used to reduce the production cost in addition to reducing and disposing of waste in a way that does not harm the environment. From the results, we find that it was possible to produce the enzyme in all types of tested oily waste, where the optimum production was in the presence of Potato oil followed by motor oil. Vegetable frying oil, chicken and fish frying oil^58^ produced the lipase enzyme by *A. niger* using the oily waste of the palm oil.

The optimal time for production was also studied. It was found that it is also 3 days, where an increase in the period caused a significant decrease in the enzymatic activity as a result of the critical diminishing nutrients present in the environment by increasing the period and not being compensated, in addition to the secondary metabolism considered to be inhibiting enzyme production. Therefore, Industrial waste is a promising candidate for use in the industrial applications involved in enzyme production and biotechnological transformations^3^.

The optimum conditions for enzyme activity and stability were determined. Then the possibility of its application in various applications was tested. The effectiveness of the enzyme and its activity in the presence of powder and liquid commercial detergents was studied at a concentration of 0.1% and 1%, as the lipase enzymes used in detergents need to be active and stable in Alkaline environments (pH 8–11) you encounter in severe washing conditions. Among the results obtained, it was found that in the presence of powder detergents, the concentration of 0.1% of the Omo powder for the enzyme produced by *A. niger* MH078571.1, and 1% of Ariel A. *A. niger* MH079049.1, while the liquid detergent 0.1% Ferry was the most appropriate. To maintain the enzymatic activity, while Bacha et al.^30^ found that the activity of the enzyme ALA1 was 100% in the presence of Ariel for powder detergents, while Dac was the optimal liquid detergent for the activity of the enzyme and its activity was the best when compared with the commercial enzyme Lipolase in the presence of Various detergents, as lipase KM12 maintained 95% of its activity in the presence of many commercial detergents^29^.

These results also agreed with Das et al.^31^ where it tested the enzyme's ability to clean the peanut oil stain used for deep frying. Das et al.^31^ emphasized that the enzyme was able to enhance the ability of detergents to remove stains; however, they stated that the effectiveness of removing oil stains in the presence of detergent and enzyme was the same in cold and hot water.

A previous study concluded that a lipase produced by *Fusarium oxysporum* increased the cleaning efficacy with various commercial detergents^59^. Hemachander and Puvanakrishnan^60^ also confirmed that the presence of detergents with a lipase produced by *Ralstonia pickettii* increased the effectiveness of removing stains by 24–27% compared to its treatment with only detergents.

Fat biodegradation is a critical characteristic of lipase enzymes. It was found that analytic enzymes such as lipase enzymes can solve environmental issues of fat pollutants in a safer and cheaper way^61^. In this study, the lipolysis property was studied by the lipase Grease both chicken and sheep to know the ability of the enzyme to Lipolysis fats in each of them and found that the enzyme managed to Lipolysis the fat mass within 6 days of incubation at optimal temperatures for each enzyme, while the enzyme produced by *L. plantarum* managed to lipolysis fats within 3 days^33^, from the results obtained, this enzyme can be applied to removing fats in the medical field as well as Lipolysis fats in water Sanitation and water pollution prevention^33^.

In this study, Biochemical characterizations of lipase enzyme activity and stability for the two highest lipase producer strains were examined *A. niger* MH078571.1 and *A. niger* MH079049.1*.* Lipase production of two isolates was studied on medium contains waste oil. The optimal conditions for lipase activity were as follows: 50% of acetone as organic solvents, 0.1% of tween 80 as surfactants. Furthermore, mg^2+^ and zn^2+^ ions enhanced lipase activity of *A. niger* MH078571.1 while Na^2+^ and cu^2+^ enhanced enzyme activity of *A. niger* MH079049.1. Lipase activity was tested for industrial applications such as integrating the enzyme with different detergents. Moreover, animal natural animal fat degradation with crude enzyme was tested using chicken and sheep fats.

As a result of these findings, the crude fungal lipase produced by both *A. niger* MH078571.1 and *A. niger* MH079049.1 strains has been purified. The purified lipases were tested to identify their molecular sizes and properties. This project, which is currently in progress, aims to determine purified lipases’ sequences and related genes through BLAST.

## Supplementary Information

Supplementary Information.

## Data Availability

The following information was supplied regarding data availability: The raw measurements are available in the Supplemental Information.
