# Supplementary material for: Application and characterization of crude fungal lipases used to degrade fat and oil wastes
Source: Sci Rep. 2021 Oct 4;11:19670. doi: 10.1038/s41598-021-98927-4 (PMC8490430; doi:10.1038/s41598-021-98927-4)
Supplement: Supplementary file 1 — Supplementary Information. [file 41598_2021_98927_MOESM1_ESM.docx]

**Supplements data Tables**

Article Type: Original Article

Application and Characterization of Crude Fungal Lipases Used to Degrade Fat and Oil Wastes

Amira H. Alabdalall^1^, Norah A. Al-Anazi^1^, Lena A. Aldakheel^1^, Fatma H.I. Amer^2^, Fatimah A Aldakheel^3^, Ibtisam M Ababutain^1,3^, Azzah I Alghamdi^1,3^, Eida M Al-Khaldi^1,3^

^1^Department of Biology, College of Science, Imam Abdulrahman Bin Faisal University, Dammam, Saudi Arabia

^2^Family and Community Medicine Department, College of Medicine, Imam Abdulrahman Bin Faisal University

^3^Department of Chemistry, College of Science, Imam Abdulrahman Bin Faisal University, Dammam, Saudi Arabia

*Corresponding Author

Amira Hassan Alabdalall

Biology Department

Imam Abdulrahman Bin Faisal University

Dammam, P.O.Box 1982

00966504822960

aalabdalall@iau.edu.sa

**Study of the Biochemical Characteristic for the lipase enzyme**

Table S1 The effect of temperature on lipase activity and stability for A. niger MH078571.1 and MH079049.1

| **Time** | **1 hours** | **2 hours** | **4 hours** | **6 hours** | **24 hours** |
| --- | --- | --- | --- | --- | --- |
| **A. niger MH078571.1** | | | | | |
| **25 ºC** | 482.31  ±9.79 | 498.85  ±7.07 | 410.39  ±5.98 | 385.39  ±5.44 | 365.77  ±1.63 |
| **30 ºC** | 500.00  ±11.97 | 488.08  ±12.51 | 447.7  ±18.49 | 409.62  ±4.90 | 371.92  ±10.33 |
| **35 ºC** | 534.23  ±4.90 | 511.54  ±4.35 | 491.54  ±11.97 | 469.62  ±8.16 | 444.62  ±7.61 |
| **40 ºC** | 734.62  ±33.72 | 692.7  ±5.98 | 623.85  ±27.20 | 581.92  ±8.16 | 481.54  ±17.41 |
| **45 ºC** | 794.23  ±5.98 | 756.16  ±18.49 | 716.16  ±7.62 | 684.2  ±0.54 | 668.46  ±9.79 |
| **50 ºC** | 712.31  ±10.88 | 641.16  ±46.23 | 554.62  ±5.44 | 516.92  ±4.35 | 486.15  ±1.09 |
| **55 ºC** | 676.54  ±7.07 | 568.08  ±0.54 | 535.00  ±0.54 | 426.54  ±16.86 | 356.92  ±5.44 |
| **60 ºC** | 597.69  ±4.35 | 534.62  ±2.18 | 499.62  ±0.54 | 390.77  ±3.26 | 143.85  ±9.79 |
| **65 ºC** | 581.54  ±6.53 | 488.08  ±15.77 | 323.85  ±10.88 | 270.77  ±4.35 | 24.62  ±2.18 |
| **70 ºC** | 424.62  ±2.18 | 258.47  ±14.14 | 173.08  ±1.09 | 30.39  ±9.25 | 5.00  ±1.63 |
| ***A. niger* MH079049.1** | | | | | |
| **25 ºC** | 414.23  ±11.42 | 516.15  ±6.53 | 493.08  ±1.09 | 474.61  ±5.44 | 441.54  ±1.09 |
| **30 ºC** | 443.08  ±1.09 | 545.77  ±0.54 | 536.54  ±1.63 | 573.08  ±18.49 | 505.77  ±2.72 |
| **35 ºC** | 613.08  ±2.18 | 590.77  ±1.09 | 570.00  ±2.18 | 555.39  ±1.09 | 525.00  ±2.72 |
| **40 ºC** | 685.39  ±1.09 | 607.69  ±4.35 | 597.70  ±2.18 | 579.62  ±0.54 | 548.46  ±1.09 |
| **45 ºC** | 735.77  ±17.95 | 681.92  ±0.54 | 586.92  ±3.26 | 575.00  ±0.54 | 556.92  ±1.09 |
| **50 ºC** | 753.46  ±16.86 | 718.08  ±24.48 | 690.00  ±1.09 | 687.31  ±0.54 | 566.15  ±4.35 |
| **55 ºC** | 796.92  ±3.26 | 770.39  ±0.54 | 763.85  ±1.09 | 717.69  ±3.26 | 684.62  ±1.09 |
| **60 ºC** | 413.85  ±1.09 | 326.54  ±8.16 | 233.85  ±1.09 | 172.31  ±8.70 | 101.15  ±0.54 |
| **65 ºC** | 317.31  ±5.98 | 216.16  ±3.26 | 158.46  ±4.35 | 116.92  ±8.70 | 28.08  ±3.81 |
| **70 ºC** | 268.46  ±11.97 | 150.77  ±2.18 | 117.31  ±1.63 | 87.31  ±3.81 | 6.54  ±2.72 |

* Averages of three replicates

Table S2 The effect of pH on lipase activity and stability for *A. niger* MH078571.1 and MH079049.1

| **Time** | **1 hours** | **2 hours** | **4 hours** | **6 hours** | **24 hours** |
| --- | --- | --- | --- | --- | --- |
| ***A. niger* MH078571.1** | | | | | |
| **pH3** | 182.69  ±0.55 | 128.85  ±0.54 | 72.308  ±6.53 | 16.539  ±0.54 | 4.231  ±0.54 |
| **pH4** | 198.85  ±0.55 | 150  ±1.09 | 86.539  ±4.90 | 33.077  ±5.44 | 6.538  ±2.72 |
| **pH5** | 259.62  ±0.55 | 243.47  ±1.63 | 209.62  ±0.54 | 193.08  ±5.44 | 170.00  ±16.32 |
| **pH6** | 441.92  ±0.55 | 425.77  ±0.54 | 401.94  ±5.98 | 357.7  ±4.35 | 331.16  ±9.25 |
| **pH7** | 718.46  ±1.09 | 689.23  ±10.88 | 608.08  ±1.63 | 557.69  ±15.23 | 499.62  ±11.42 |
| **pH8** | 795.39  ±0.55 | 776.54  ±0.54 | 748.47  ±3.26 | 720.00  ±10.88 | 683.84  ±1.09 |
| **pH9** | 727.69  ±10.88 | 673.84  ±10.88 | 604.62  ±21.76 | 486.54  ±7.07 | 419.23  ±5.44 |
| **pH10** | 688.46  ±1.09 | 588.07  ±0.54 | 504.62  ±10.88 | 408.46  ±5.44 | 337.69  ±9.79 |
| ***A. niger* MH079049.1** | | | | | |
| **pH3** | 196.92  ±3.26 | 184.23  ±23.39 | 149.62  ±0.54 | 68.07  ±0.54 | 4.23  ±0.54 |
| **pH4** | 328.85  ±0.54 | 299.62  ±3.81 | 150.00  ±1.09 | 121.54  ±0.00 | 28.08  ±0.54 |
| **pH5** | 414.62  ±1.09 | 386.92  ±3.26 | 386.92  ±3.26 | 358.46  ±0.00 | 243.08  ±10.88 |
| **pH6** | 491.53  ±7.62 | 459.62  ±9.25 | 432.31  ±4.35 | 406.16  ±1.09 | 364.62  ±10.88 |
| **pH7** | 688.46  ±9.79 | 644.23  ±52.76 | 596.54  ±17.95 | 573.08  ±5.44 | 533.85  ±2.18 |
| **pH8** | 795.77  ±1.09 | 761.15  ±4.90 | 740.77  ±1.09 | 701.92  ±0.54 | 636.16  ±9.79 |
| **pH9** | 673.85  ±43.51 | 593.84  ±6.53 | 563.85  ±1.09 | 529.62  ±0.54 | 507.70  ±0.54 |
| **pH10** | 575.77  ±1.63 | 577.69  ±0.54 | 548.46  ±0.54 | 489.62  ±0.54 | 415.00  ±0.54 |

* Averages of three replicates

Table S3 The effect of different organic solvents on both *A. niger* MH078571.1 and MH079049.1 lipase activity and stability

| **Strain** | ***A. niger* MH078571.1** | | | | ***A. niger* MH079049.1** | | | | |
| --- | --- | --- | --- | --- | --- | --- | --- | --- | --- |
| **Concentration** | **100%** | | **50%** | | **100%** | | | **50%** | |
|  | **Lipase activity (U/ml)** | **Percent**  **(%)** | **Lipase activity (U/ml)** | **Percent**  **(%)** | | **Lipase activity (U/ml)** | **Percent (%)** | **Lipase activity (U/ml)** | **Percent (%)** |
| **Negative Control** | 745.00  ± 7.07 | 100 | 745.00  ± 7.07 | 100 | | 741.54  ± 2.18 | 100 | 741.54  ± 2.18 | 100 |
| **Methanol** | 575.38  ± 10.33 | 77.23 | 621.15  ± 21.76 | 83.38 | | 449.62  ± 9.25 | 60.63 | 551.92  ± 3.81 | 74.43 |
| **Eethanol** | 254.62  ± 12.51 | 34.18 | 414.23  ± 4.35 | 55.62 | | 367.70  ± 32.64 | 49.59 | 470.00  ± 9.79 | 63.38 |
| **Isopropanol** | 306.16  ± 5.44 | 41.1 | 377.69  ± 5.44 | 50.7 | | 330.00  ± 8.16 | 44.50 | 428.85  ± 18.49 | 57.83 |
| **Butanol** | 493.85  ± 17.41 | 66.29 | 563.85  ± 16.32 | 75.68 | | 312.31  ± 24.48 | 42.12 | 576.54  ± 38.08 | 77.75 |
| **Aceton** | 586.54  ± 10.89 | 78.73 | 719.23  ± 3.81 | 96.54 | | 260.77  ± 23.39 | 35.17 | 690.39  ± 17.41 | 93.10 |

* Averages of three replicates

Table S4 The effect of different surfactants on both *A. niger* MH078571.1 and MH079049.1 lipase activity and stability

| **Strain** | ***A. niger* MH078571.1** | | | | ***A. niger* MH079049.1** | | | | |
| --- | --- | --- | --- | --- | --- | --- | --- | --- | --- |
| **Concentration** | **1%** | | **0.1%** | | **1%** | | | **0.1%** | |
|  | **Lipase activity (U/ml)** | **Percent**  **(%)** | **Lipase activity (U/ml)** | **Percent**  **(%)** | | **Lipase activity (U/ml)** | **Percent (%)** | **Lipase activity (U/ml)** | **Percent (%)** |
| **Negative Control** | 764.62  ± 10.89 | 100 | 764.62  ± 10.89 | 100 | | 743.08  ± 3.26 | 100 | 740  ± 3.26 | 100 |
| **SDS** | 250.77  ± 34.81 | 32.8 | 511.92  ± 8.16 | 66.95 | | 471.92  ± 14.69 | 63.51 | 414.2  ± 0.54 | 55.98 |
| **Tween 80** | 433.08  ± 10.88 | 56.64 | 790.77  ± 2.19 | 103.42 | | 496.54  ± 15.77 | 66.82 | 831.92  ± 15.77 | 112.4 |
| **Tween 20** | 174.62  ± 9.79 | 22.84 | 541.15  ± 3.81 | 70.77 | | 263.08  ± 23.93 | 35.40 | 469.23  ± 25.02 | 63.40 |

* Averages of three replicates

Table S5 The effect of different ions on *A. niger* MH078571.1 lipase activity

| **Strain** | ***A. niger* MH078571.1** | | | | ***A. niger* MH079049.1** | | | | |
| --- | --- | --- | --- | --- | --- | --- | --- | --- | --- |
| **Concentration** | **1%** | | **0.1%** | | **1%** | | | **0.1%** | |
|  | **Lipase activity (U/ml)** | **Percent**  **(%)** | **Lipase activity (U/ml)** | **Percent**  **(%)** | | **Lipase activity (U/ml)** | **Percent (%)** | **Lipase activity (U/ml)** | **Percent (%)** |
| **Negative Control** | 728.08  ± 20.13 | 100 | 728.08  ± 20.13 | 100 | | 733.08  ± 38.08 | 100 | 733.08  ± 38.08 | 100 |
| **NaCl** | 426.15  ± 19.58 | 58.53 | 596.15  ± 27.20 | 81.88 | | 251.54  ± 6.53 | 34.31 | 769.62  ± 27.74 | 104.98 |
| **CaCl_2_** | 356.92  ± 9.79 | 49.02 | 465.77  ± 1.63 | 63.97 | | 256.15  ± 11.97 | 34.94 | 349.62  ± 0.54 | 47.69 |
| **KCl** | 388.85  ± 5.98 | 53.41 | 429.62  ± 12.51 | 59.01 | | 347.69  ± 9.79 | 47.43 | 375.00  ± 17.95 | 51.154 |
| **NH_4_Cl** | 479.23  ± 5.44 | 65.82 | 518.85  ± 11.42 | 71.26 | | 413.85  ± 9.79 | 56.46 | 493.85  ± 13.05 | 67.37 |
| **MgSO_4_** | 643.46  ± 27.74 | 88.38 | 743.46  ± 61.46 | 102.11 | | 590.77  ± 5.44 | 80.59 | 586.92  ± 19.58 | 80.06 |
| **ZnSO_4_** | 684.23  ± 21.21 | 93.98 | 743.46  ± 31.00 | 102.11 | | 506.54  ± 1.63 | 69.1 | 596.16  ± 16.31 | 81.32 |
| **CuSO_4_** | 638.462  ± 27.2 | 87.69 | 724.62  ± 5.44 | 99.53 | | 497.7  ± 19.58 | 67.89 | 685.00  ± 78.87 | 93.44 |
| **EDTA** | 431.54  ± 11.97 | 59.27 | 514.23  ± 3.81 | 70.63 | | 511.54  ± 13.05 | 69.78 | 463.08  ± 15.23 | 63.17 |

* Averages of three replicates

Table S6 The determination of the optimal storage temperature for both A. niger MH078571.1 and MH079049.1 lipase activity and stability

| **Time** | **Initial** | | **1 week** | | **2 weeks** | | **3 weeks** | | **4 weeks** | |
| --- | --- | --- | --- | --- | --- | --- | --- | --- | --- | --- |
| **Temp.** | Lipase activity (U/ml) | Stability (%) | Lipase activity (U/ml) | Stability (%) | Lipase activity (U/ml) | Stability (%) | Lipase activity (U/ml) | Stability (%) | Lipase activity (U/ml) | Stability (%) |
| *A. niger* MH078571.1 | | | | | | | | | | |
| **25 ºC** | 756.92  ±16.32 | 100 | 399.62  ±2.72 | 52.8 | 263.08  ±2.18 | 34.76 | 116.54  ±7.07 | 15.4 | 34.231  ±1.63 | 4.5 |
| **4 ºC** | 756.92  ±16.32 | 100 | 589.62  ±8.16 | 77.9 | 500.39  ±12.51 | 66.11 | 406.54  ±0.54 | 53.71 | 359.23  ±2.18 | 47.46 |
| **-20 ºC** | 756.92  ±16.32 | 100 | 680.38  ±1.63 | 89.89 | 583.08  ±7.61 | 77.03 | 543.47  ±4.90 | 71.8 | 389.23  ±10.88 | 51.42 |
| **-80 ºC** | 756.92  ±16.32 | 100 | 687.31  ±1.63 | 90.8 | 678.47  ±15.23 | 89.64 | 598.08  ±2.72 | 79.02 | 375.00  ± 17.95 | 51.154 |
| *A. niger* MH079049.1 | | | | | | | | | | |
| **25 ºC** | 756.16  ±5.44 | 100 | 322.69  ±9.25 | 42.68 | 198.85  ±10.33 | 26.30 | 87.308  ±8.16 | 11.55 | 15.00  ±7.07 | 1.98 |
| **4 ºC** | 756.15  ±5.44 | 100 | 598.85  ±11.42 | 79.20 | 464.62  ±18.49 | 61.44 | 410.77  ±5.44 | 54.32 | 270.39  ±0.54 | 35.76 |
| **-20 ºC** | 756.15  ±5.44 | 100 | 694.23  ±14.69 | 91.81 | 526.92  ±15.23 | 69.69 | 448.46  ±13.05 | 59.31 | 408.46  ±21.76 | 54.02 |
| **-80 ºC** | 756.15  ±5.44 | 100 | 743.85  ±9.79 | 98.37 | 682.31  ±1.09 | 90.23 | 585.38  ±8.70 | 77.42 | 552.31  ±1.09 | 73.04 |

* Averages of three replicates

Table S7 The determination of lipase efficiency and activity in the present of natural oil waste in both A. niger isolates

| **Time** | **3 days** | **5 days** | **7 days** | **10 days** | **15 days** |
| --- | --- | --- | --- | --- | --- |
| **Vehichles oil** | | | | | |
| *A. niger MH078571.1* | 641.28  ±41.13 | 553.33  ±5.88 | 377.69  ±18.09 | 262.05  ±8.75 | 225.39  ±7.99 |
| *A. niger MH079049.1* | 621.28  ±41.21 | 551.54  ±11.38 | 319.49  ±8.47 | 309.49  ±17.48 | 266.16  ±16.55 |
| **Fish fry oil** | | | | | |
| *A. niger MH078571.1* | 483.59  ±6.94 | 378.21  ±18.03 | 96.41  ±3.47 | 68.72  ±14.72 | 52.82  ±7.74 |
| *A. niger MH079049.1* | 474.87  ±17.34 | 236.67  ±12.34 | 192.05  ±23.49 | 137.44  ±15.85 | 84.87  ±15.79 |
| **Chicken fry oil** | | | | | |
| *A. niger MH078571.1* | 496.15  ±19.05 | 367.69  ±18.48 | 239.23  ±21.70 | 202.57  ±6.72 | 121.28  ±12.27 |
| *A. niger MH079049.1* | 498.97  ±15.62 | 266.67  ±16.58 | 192.31  ±15.61 | 179.23  ±20.65 | 124.87  ±7.71 |
| **Potato fry oil** | | | | | |
| *A. niger MH078571.1* | 666.31  ±49.66 | 273.33  ±6.54 | 220  ±14.86 | 126.15  ±7.34 | 32.56  ±1.60 |
| *A. niger MH079049.1* | 706.41  ±18.91 | 375.64  ±21.18 | 185.39  ±15.37 | 124.10  ±4.95 | 48.46  ±9.23 |
| **Vegetables fry oil** | | | | | |
| *A. niger MH078571.1* | 581.28  ±16.03 | 523.33  ±20.77 | 197.95  ±25.77 | 121.79  ±12.93 | 41.28  ±16.16 |
| *A. niger MH079049.1* | 642.31  ±20.22 | 574.62  ±33.18 | 273.33  ±6.63 | 235.9  ±10.94 | 198.46  ±1.33 |

* Averages of three replicates

Table S8 The determination of lipase efficiency and activity of both A. niger MH079049 and MH078571 on powder detergent

| **Concentration of Powder detergent** | **Lipase activity**  **(U/ml)** | **Percent**  **%** | **Lipase activity**  **(U/ml)** | **Percent**  **%** |
| --- | --- | --- | --- | --- |
| **Strain** | A. niger *MH079049* | | A. niger *MH078571* | |
| **Control** | 645.9 | 100% | 689.23 | 100% |
| **Tide** | | | | |
| **0.1%** | 598.72 | 92.70% | 577.69 | 83.82% |
| **1%** | 410 | 63.48% | 239.74 | 34.78% |
| **Arial** | | | | |
| **0.1%** | 392.57 | 60.78% | 118.21 | 17.15% |
| **1%** | 591.8 | 91.62% | 559.49 | 81.18% |
| **Pandah** | | | | |
| **0.1%** | 590.77 | 91.46% | 424.10 | 61.53% |
| **1%** | 291.54 | 45.14% | 122.82 | 17.82% |
| **Persil** | | | | |
| **0.1%** | 478.46 | 74.08% | 481.54 | 69.87% |
| **1%** | 614.10 | 95.08% | 581.28 | 84.34% |
| **Omo** | | | | |
| **0.1%** | 39.744 | 6.15% | 46.923 | 6.81% |
| **1%** | 582.82 | 90.23% | 627.69 | 91.07% |

* Averages of three replicates

Table S9 The determination of lipase efficiency and activity of both A. niger MH078571 and MH079049 on liquid detergent

| **Concentration of Liquid detergent** | **Lipase activity**  **(U/ ml)** | **Percent**  **%** | **Lipase activity**  **(U/ ml)** | **Percent**  **%** |
| --- | --- | --- | --- | --- |
| **Strain** | A. niger *MH078571* | | A. niger *MH079049* | |
| **control** | 689.23 | 100% | 645.9 | 100% |
| **Fairy** | | | | |
| **0.1%** | 640.26 | 92.89% | 577.43 | 89.40% |
| **1%** | 392.31 | 56.92% | 119.23 | 18.46% |
| **Pandah** | | | | |
| **0.1%** | 404.62 | 58.71% | 432.05 | 66.89% |
| **1 %** | 293.84 | 42.63% | 231.03 | 35.77% |
| **Dac** | | | | |
| **0.1%** | 330.77 | 47.99% | 493.85 | 76.46% |
| **1 %** | 388.97 | 56.44% | 233.08 | 36.09% |
| **Lux** | | | | |
| **0.1%** | 621.03 | 90.10% | 261.8 | 40.53% |
| **1 %** | 550 | 79.80% | 630 | 97.54% |
| **Perial** | | | | |
| **0.1%** | 171.28 | 24.85% | 257.44 | 39.86% |
| **1 %** | 257.18 | 37.31% | 200 | 30.96% |

* Averages of three replicates

**Study of the Biochemical Characteristic for the lipase enzyme**

| **(A)** |
| --- |
| **(B)** |

Fig. S1: The effect of temperature on lipase activity and stability for both A. niger **(A)** MH078571.1 and **(B)** MH079049.1

| **(A)** |
| --- |
| **(B)** |

Fig. S2: The effect of pH on lipase activity and stability for both A. niger **(A)** MH078571.1 and **(B)** MH079049.1

| **(A)** |
| --- |
| **(B)** |

Fig S3: The effect of different organic solvents on lipase activity and stability for both A. niger **(A)** MH078571.1 and **(B)** MH079049.1

| **(A)** |
| --- |
| **(B)** |

Fig S4: The effect of different surfactants on lipase activity and stability for both A. niger **(A)** MH078571.1 and **(B)** MH079049.1

| **(A)** |
| --- |
| **(B)** |

Fig S5: The effect of different ions on lipase activity for both *A. niger* **(A)** MH078571.1 and **(B)** MH079049.1

| **(A)** |
| --- |
| **(B)** |

Fig S6: The determination of the optimal storage temperature for lipase activity and stability on both A. niger **(A)** MH078571.1 and **(B)** MH079049.1

| **(A)** |
| --- |
| **(B)** |

Fig S7: The determination of lipase efficiency and activity for A. niger MH078571 on both **(A)** powder detergents and **(B)** liquid detergents.

| **(A)** |
| --- |
| **(B)** |

Fig S8: The determination of lipase efficiency and activity for A. niger MH079049 on both **(A)** powder detergents and **(B)** liquid detergents.

| **(A)** |
| --- |
| **(B)** |

Fig S9: The determination of lipase efficiency and activity in removing oil stain from polycotton fabric for both *A. niger* **(A)** MH078571.1 and **(B)** MH079049.1

| ***Stains*** | **Vehicles oil** | | **Fish oil** | | **Chocolate stain** | |
| --- | --- | --- | --- | --- | --- | --- |
| **Strain**  **Treatment** | ***A. niger* MH078571.1** | ***A. niger* MH079049.1** | ***A. niger* MH078571.1** | ***A. niger* MH079049.1** | ***A. niger* MH078571.1** | ***A. niger* MH079049.1** |
| **Hot water + detergent** | 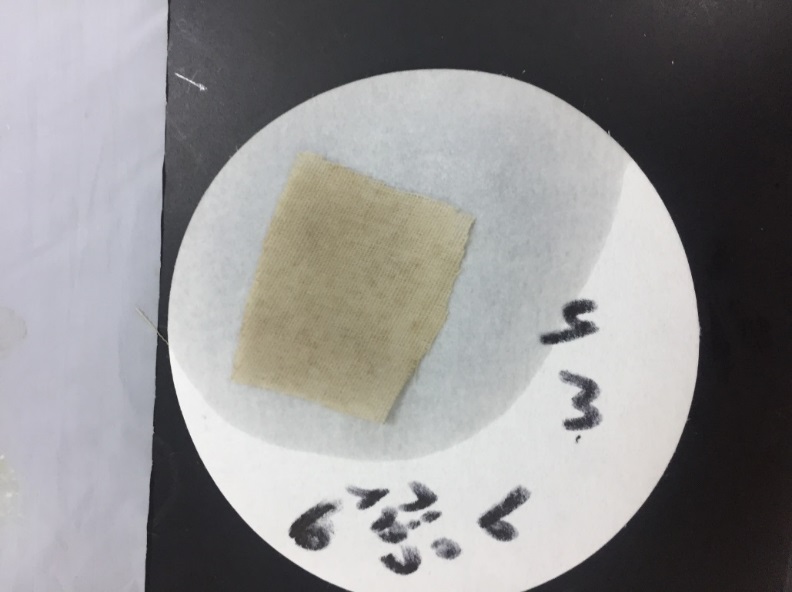 | 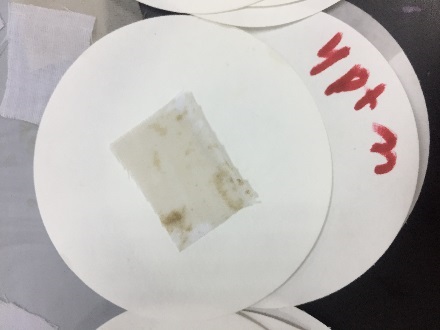 | 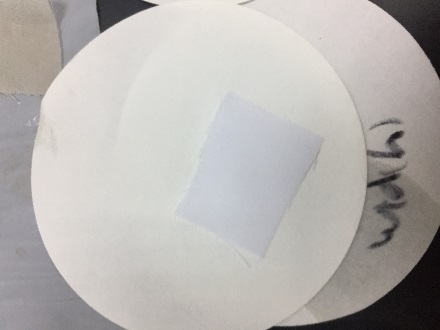 | 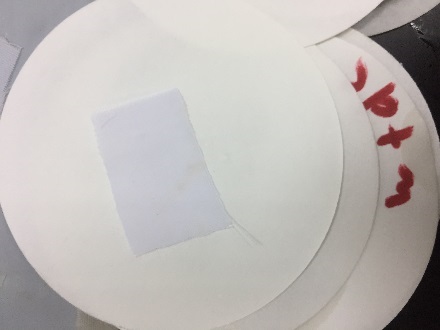 | 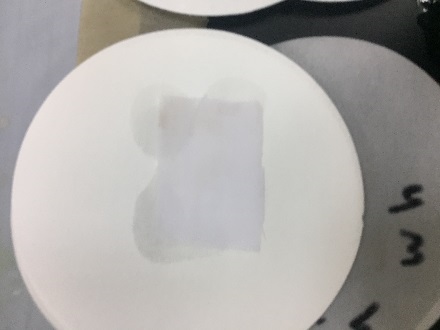 | 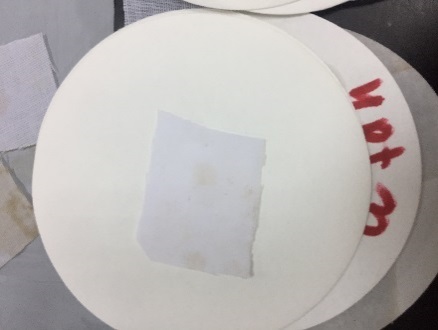 |
| **Hot water + lipase** | 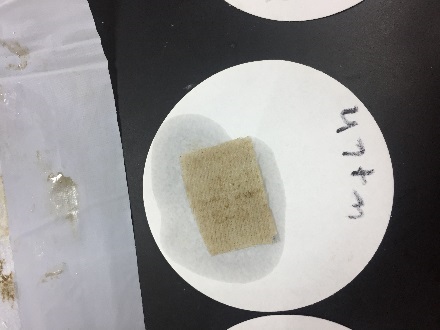 | 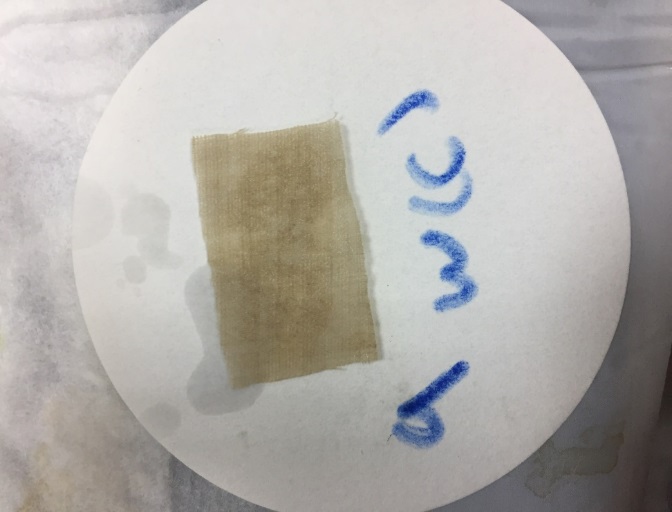 | 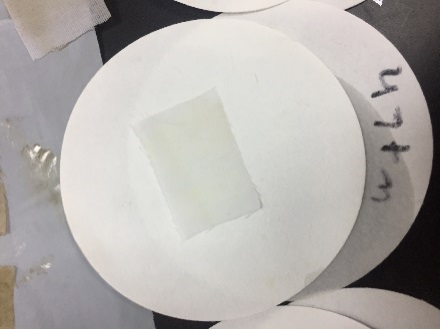 | 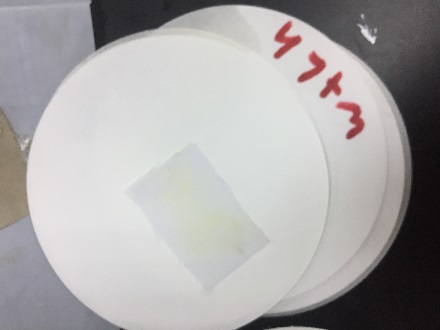 | 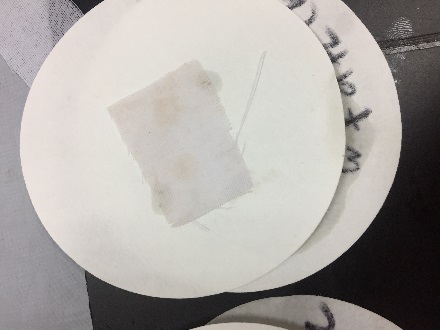 | 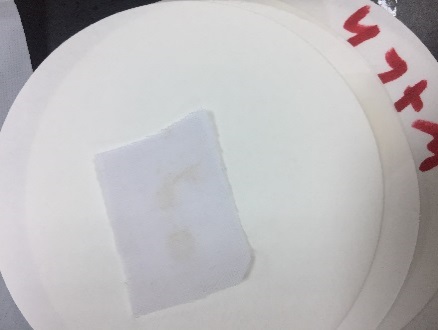 |
| **Cold water + detergent** | 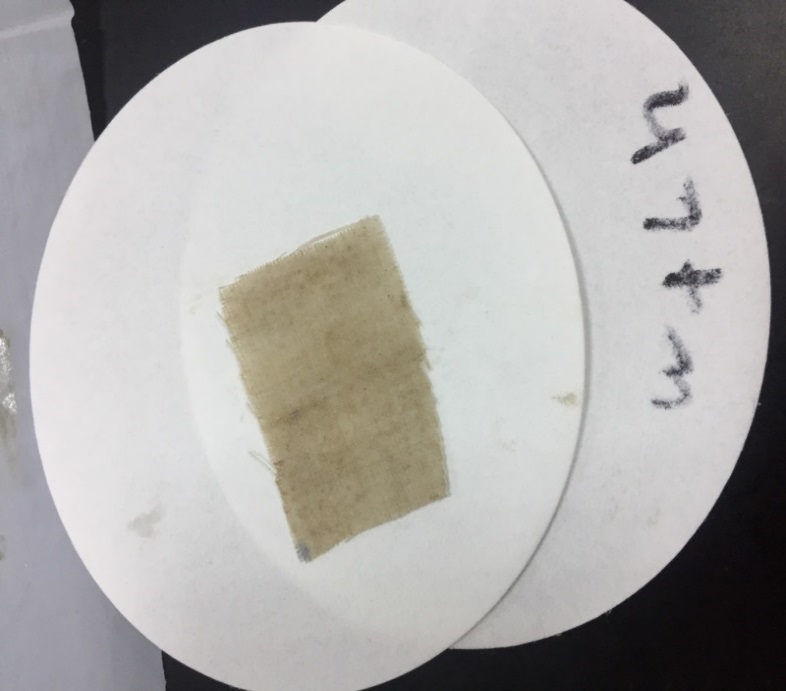 | 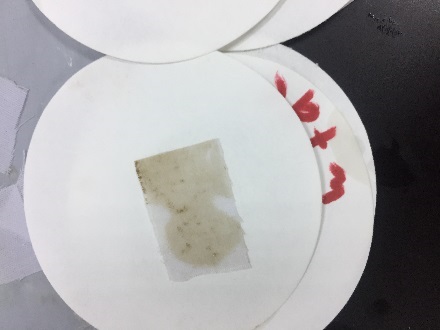 | 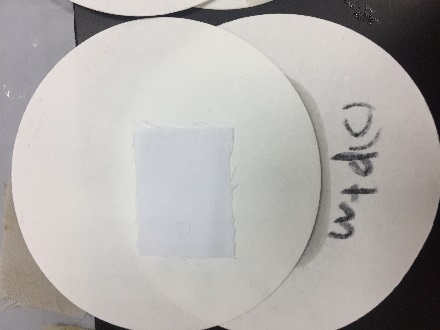 | 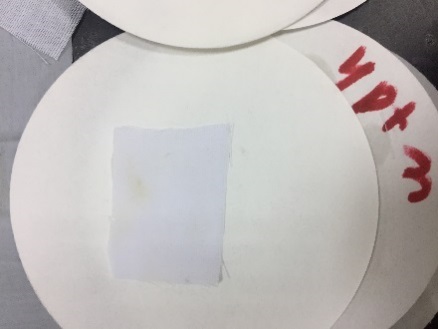 | 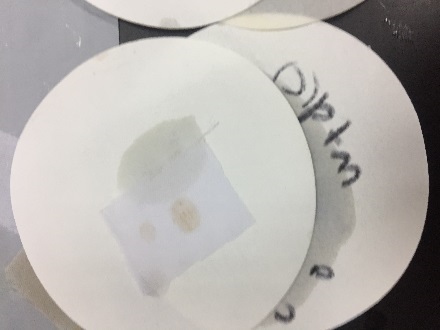 | 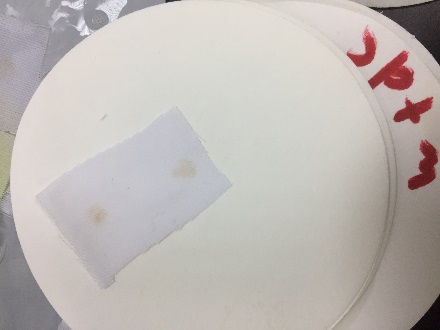 |
| Cold water + lipase | 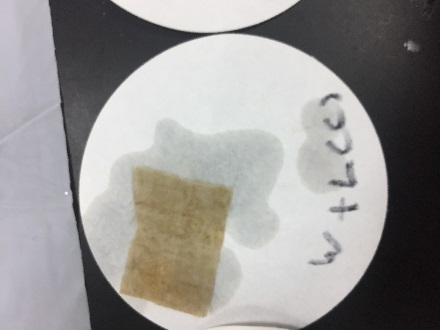 | 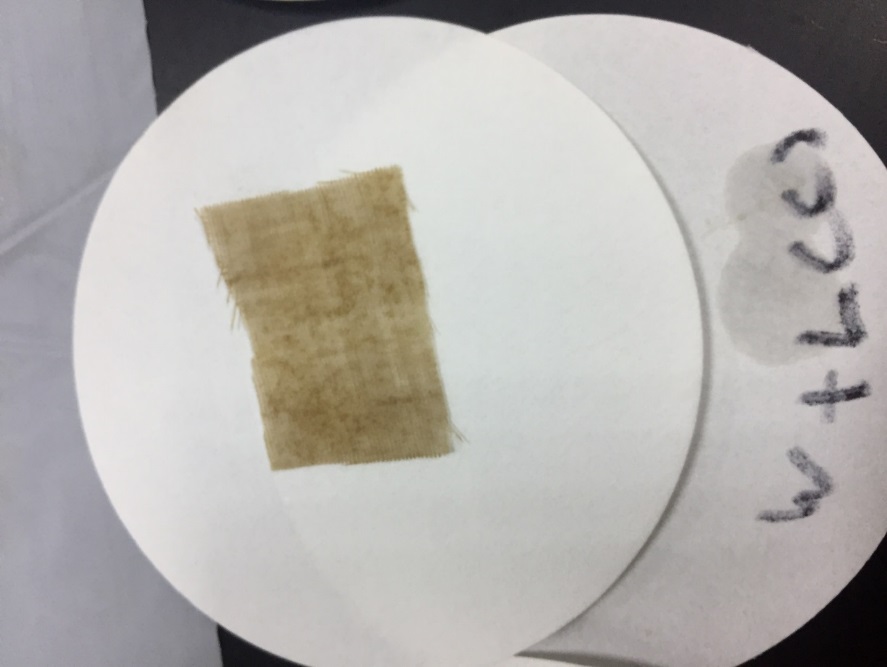 | 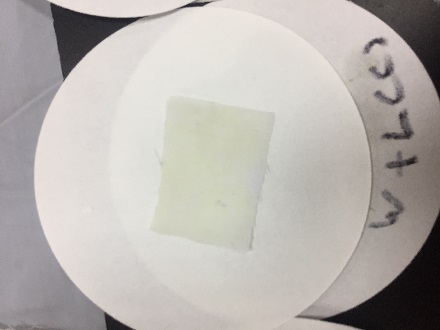 | 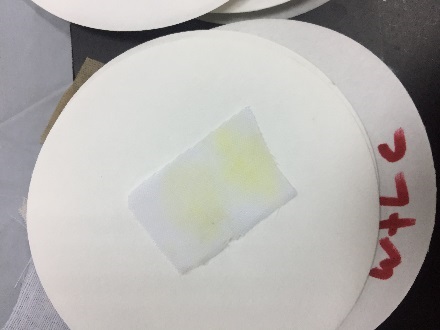 | 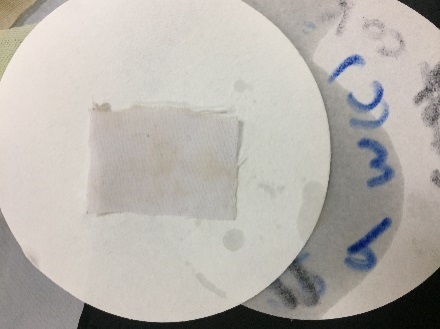 | 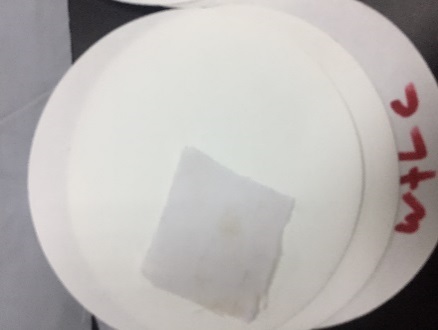 |

Fig S10 The effect lipase enzyme from both *A. niger* MH078571.1 and *A. niger* MH079049.1 on removing vehicles oil stain, fish oil stain, and chocolate stain.

| **(A)** |
| --- |
| **(B)** |

Fig S11: The determination of lipase efficiency and activity for both *A. niger* strains in degrading **(A)** chicken fat and **(B)** sheep fat

| **(A)** |
| --- |
| **(B)** |

Fig S12: The effect of lipase enzyme isolated from both *A. niger* strains on **(A)** chicken fat weight and **(B)** sheep fat weight
